# Significant boost of the stability and PLQYof CsPbBr_3_ NCs by Cu-BTC MOF

**DOI:** 10.1038/s41598-022-11874-6

**Published:** 2022-05-12

**Authors:** Hari Shankar, William W. Yu, Youngjong Kang, Prasenjit Kar

**Affiliations:** 1 Department of Chemistry, Indian Institute of Technology Roorkee, Roorkee, Uttarakhand 247667 India; 2 Department of Chemistry and Physics, Louisiana State University, Shreveport, LA 71115 USA; 3 Department of Chemistry, College of Natural Sciences, Hanyang University, 222 Wangsimni-ro, Seongdong-gu, Seoul, 04763 Korea

**Keywords:** Materials chemistry, Metal-organic frameworks

## Abstract

Developing stable perovskite nanocrystals (NCs) with enhancing luminescent properties holds great importance for future potential applications in optoelectronics. Here, we engaged perovskite NCs in Cu^2+^ ion-based metal–organic framework (MOF) Cu-BTC (BTC = 1,3,5-benzene tricarboxylate) by physical mixing of MOF with CsPbBr_3_ NCs in toluene solution. MOF-protected perovskite NCs achieved high photoluminescence quantum yield 96.51% than pristine state CsPbBr_3_ NCs (51.66%). Along with the improvement in optical properties, the long-term stability of CsPbBr_3_ NCs in the solution phase also increases considerably upon loading in Cu-BTC MOF. Moreover, the changes in the luminescent intensity of the samples have been observed for 3 months in the solution. After 1 month, pristine CsPbBr_3_ NCs lose their emission intensity 68% from the initial, while the MOF-protected CsPbBr_3_ NCs show only a 10% reduction from the initial. These results indicate that the effective passivation of Cu-BTC MOF inhibits the aggregation of NCs, protecting them from the defective atmosphere. The excellent photoluminescence findings provide a new pathway for future optoelectronic applications.

## Introduction

Since the last decade, lead halide perovskite such as CsPbX_3_ (X = Cl^−^, Br^−^, I^−^) has attracted considerableattention due to their potential applications in solar cell, light-emitting diodes (LEDs), lasers, and photodetectors^1–5^. The remarkable emission properties of CsPbX_3_ perovskite nanocrystals (NCs), displaying high photoluminescence quantum yields (PLQYs), emission wavelength tunability, short irradiative lifetime, and narrowband emission behaviour enable the optoelectronic devices with excellent performances^6–9^. Despite all this, CsPbX_3_ NCs lose their crystal identity and optical performance in the presence of moisture, air, light, and heat due to their highly ionic internal structure and low formation energy^10–13^. This environmental instability hampers their commercialization. Researchers have made efforts tirelessly to get rid of this instability problem in CsPbX_3_ NCs, by replacing the cationic or anionic part, doping of elements, capping the surface with branched ligands, and blending in inorganic or organic polymers^14–17^. Moreover, porous metal oxides matrix of Al_2_O_3_, TiO_2_, and mesoporous SiO_2_ have been used to protect the perovskite NCs from environmental stimulations^18–22^

Subsequently, the porous metal–organic frameworks (MOFs) have proven beneficial for the perovskite NCs to improve the stability and extend the new application opportunities. The tunable porosity, high specific surface area, and highly active metal center of MOFs make them suitable materials for protecting perovskite NCs^23–25^. Recently, various studies have been reported to form perovskite@MOFs nanocomposites, such as generating perovskite NCs inside the MOFs, blending the precursors of MOFs and perovskites, and mixing the pre-synthesized perovskite NCs with MOF precursors. Moreover, MOF hosts can act as a trigger to improve the luminescent properties of CsPbX_3_ NCs by host–guest interactions. Xia and co-workers^26^ enhanced the stability of CsPbX_3_ NCs generated inside the Uio-67 MOF and achieved PLQYs of 38.5%. In another report, Wang and co-workers^27^ gained up to 56% of PLQYs upon physically mixing of CsPbX_3_ into MOF-5 and improved the stability of CsPbX_3_ NCs. The confined synthesis of CH_3_NH_3_PbI_2_X NCs in the porous HKUST-1 thin film template was reported in the literature^28^. Notably, this is the first report of the colloidal phase synthesis of perovskite@MOF nanocomposites to the best of our knowledge.

**Figure 1:**
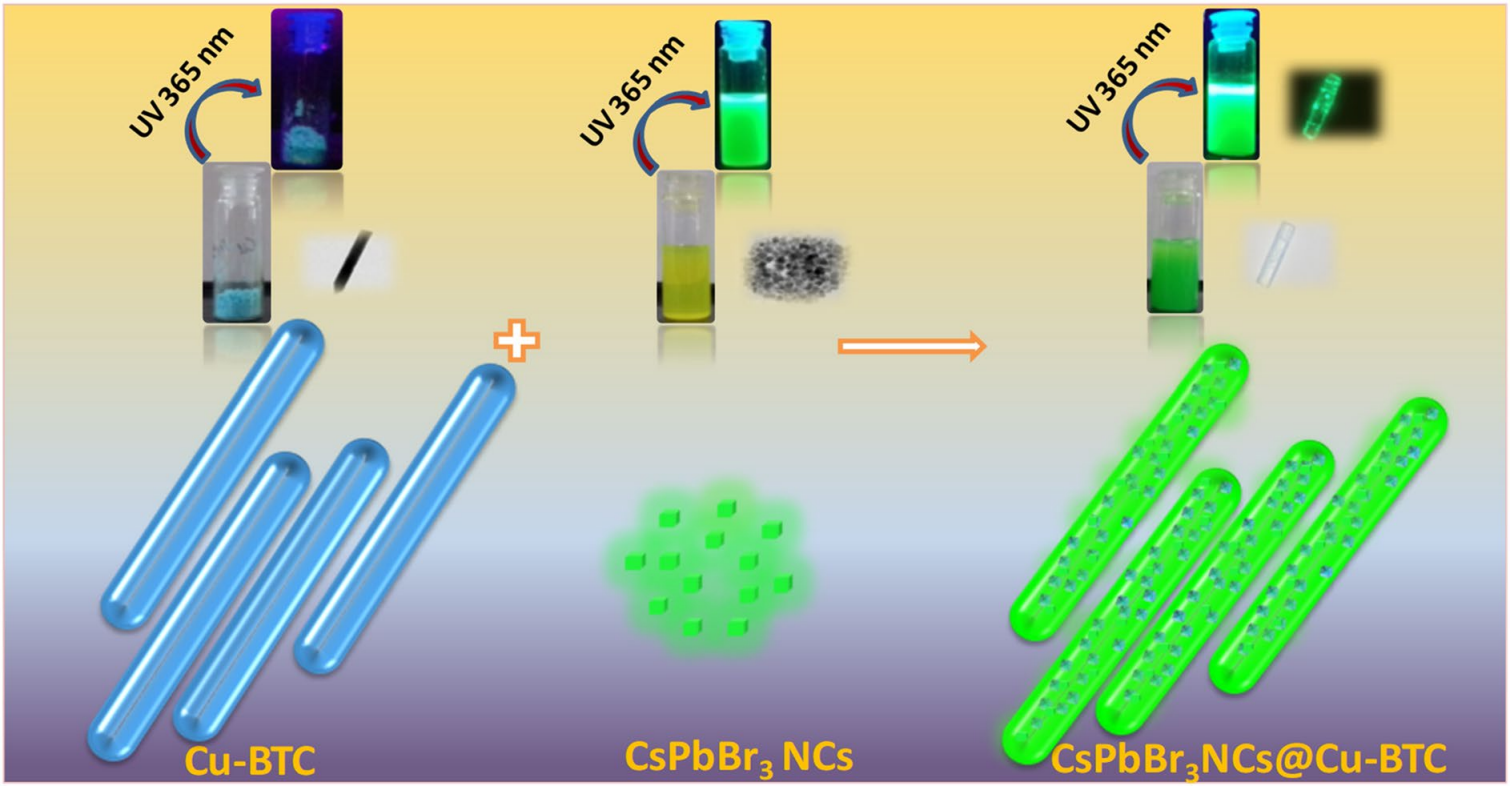
Schematic illustration for the synthesis of CsPbBr_3_ NCs@Cu-BTC composites.

Herein, we focused on Cu-1,3,5-benzene tricarboxylate (BTC) MOF as a host to prevent the degradation of pre-synthesized CsPbBr_3_ NCs. In this work, we demonstrated a straightforward strategy to incorporate the CsPbBr_3_ NCs with porous Cu-BTC MOF in the presence of toluene solvent, as shown in Fig. 1. The perovskite NCs were synthesized by the ligand-assisted re-precipitation (LARP) method.

The Cu-BTC MOF was prepared by constantly stirring aqueous copper (II) nitrate solution with ethanolic 1,3,5-benzene tricarboxylic acid (BTC) solution at RT. For the synthesis of CsPbBr_3_@Cu-BTC nanocomposite, MOF powder was added into perovskite containing toluene solvent with a constant stirring at RT. The whole synthesis process is speedy, easy, and performed in the open air at room temperature. The significantly high PLQY 96.51% was obtained in CsPbBr_3_ NCs, upon treating with Cu-MOF, while 51.66% in untreated CsPbBr_3_ NCs. The Cu-BTC MOF enhanced the PLQY and improved the chemical stability of CsPbBr_3_ NCs for months.

## Results and discussion

**Figure 2:**
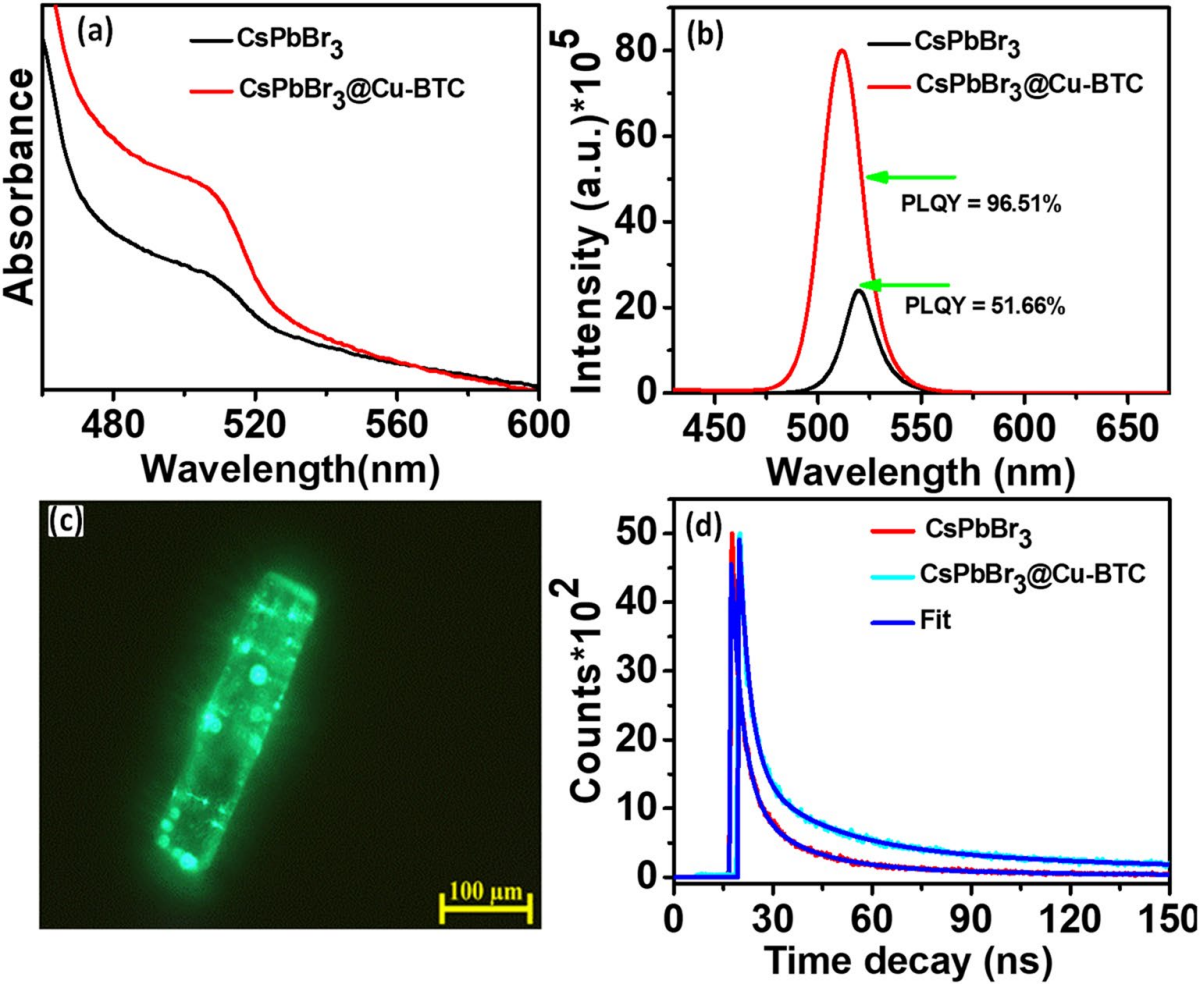
(**a**) Absorption spectra of CsPbBr_3_ NCs and CsPbBr_3_@Cu-BTC composite. (**b**) Photoluminescence spectra of CsPbBr_3_ NCs and composite. (**c**) The UV light field microscopic image of CsPbBr_3_@Cu-BTC composite. (**d**) The PL decay curves of CsPbBr_3_ NCs and CsPbBr_3_@Cu-BTC composite.

Absorption spectra (Fig. 2a) of CsPbBr_3_ NCs show a band at 509 nm and CsPbBr_3_ NCs@Cu-BTC exhibit an absorption onset at 506 nm. As shown in Fig. 2b, CsPbBr_3_ NCs display an emission peak at 519 nm in the photoluminescence spectral studies, while CsPbBr_3_ NCs@Cu-BTC shows a highly intense emission band at 512 nm. The blue shift was observed in CsPbBr_3_ NCs in both the cases absorption and emission upon loading into MOF, which may be attributed to a decrease in the size of NCs after engaging with MOF. Zhang and their co-workers have observed the blue shift in the photoluminescence spectra of MAPbI_2_Br after the formation of MAPbI_2_Br@HKUST-1 composite^28^. The tunability in optical properties is the results of quantum confinement^29^.

The PL spectrum shows full width at half maximum (FWHM) of about 18 nm for pristine CsPbBr_3_ NCs, narrower than that of guest CsPbBr_3_ NCs (23 nm). The change in FWHM suggests that the intercalating of CsPbBr_3_ NCs into the rigid porous MOF framework possibly resulted in a slight shift in particle size distribution. That meant the particle size is narrower by the intercalating process^30^. The absolute PLQYs of samples were recorded at 350 nm excitation wavelength. PLQY of 51.66% was measured in CsPbBr_3_ NCs, while 96.51% PLQY was achieved in CsPbBr_3_ NCs@Cu-BTC, hugely higher than pristine CsPbBr_3_NCs. It should be due to the more effective surface passivation of CsPbBr_3_ NCs by terminal oxygen of Cu-BTC MOF decreasing surface defect density. The improvement in PLQY as well as in emission lifetime may be due to the surface defects passivation of perovskite by Cu-BTC. In the previous reports researchers have treated the surface halide vacancies by R-COO^-^ type passivating ligand, increasing PLQY and emission lifetime of halide perovskites^31^. Pan et al. have reduced the surface trap state of perovskite NCs with 2,2′-iminodibenzoic acid achieving high PLQY and improve emission lifetime of treated perovskite NCs^32^. 96.51% PLQY of CsPbBr_3_@Cu-BTC composite is the highest PLQY than other reports for perovskite@MOF composite system^32,33^.

The Fluorescent microscopy images in Fig. 2c and Fig. S1 of CsPbBr_3_ NCs@Cu-BTC microrod show many bright green fluorescent NCs spread over the Cu-BTC rod, verifying the association of CsPbBr_3_ NCs with Cu-BTC MOF. The photoluminescence decay of samples can be described by triexponential fitting kinetics, as shown in Fig. 2d. CsPbBr_3_NCs@Cu-BTC composite was displayed a short-lived emission lifetime (τ_1_) of 4 ns, midway-lived emission lifetime (τ_2_) of 22 ns, and long-lived PL lifetime (τ_3_) of 113 ns. The average lifetime (τ_avg_) of 103 ns was observed for CsPbBr_3_ NCs@Cu-BTC, which is significantly higher than that of the average lifetime (τ_avg_) 66 ns for pristine CsPbBr_3_ NCs. The improvement in the emission lifetime of CsPbBr_3_ NCs@Cu-BTC composite should be due to the suppression of the nonradiative recombination pathway of CsPbBr_3_ NCs by Cu-BTC MOF. These results are strongly consistent with the high PLQY of the composite than pristine CsPbBr_3_ NCs.

**Figure 3:**
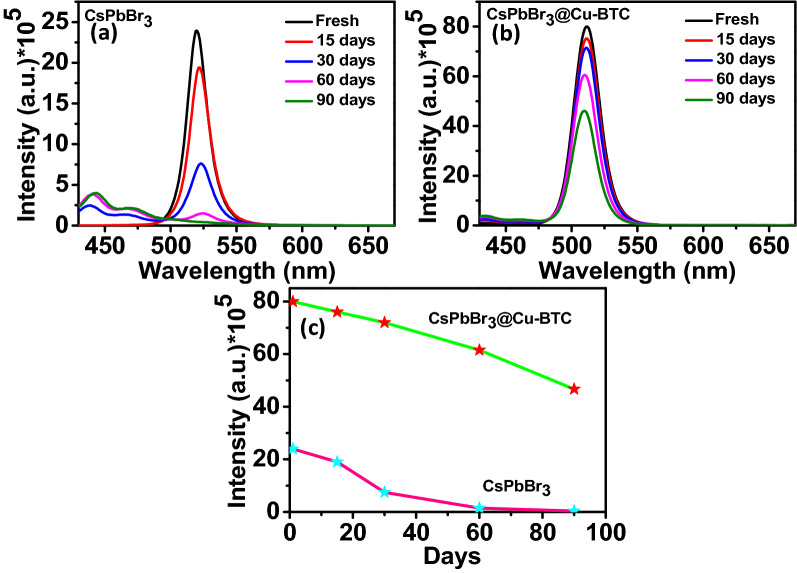
(**a**) Time-dependent emission spectra of CsPbBr_3_ NCs up to 90 days. (**b**) Time-dependent PL spectra of CsPbBr_3_@Cu-BTC composite up to 90 days. (**c**) Comparison of PL intensity decrement of CsPbBr_3_ NCs and CsPbBr_3_NCs@Cu-BTC at different days.

Furthermore, the emission behaviour of samples immersed in toluene solution was recorded for 90 days to appraise the stability of CsPbBr_3_ NCs. As shown in Fig. 3a, the fluorescence intensity decreases 68% from the first day to 30 days for pristine CsPbBr_3_ NCs, while this loss was 10% for CsPbBr_3_ NCs@Cu-BTC (Fig. 3b). In this order, after 90 days, pristine CsPbBr_3_ NCs lost 98% of initial PL intensity, and MOF-protected CsPbBr_3_ NCs still preserved 56% of their initial intensity (Fig. 3c). These results clearly show that the emission intensity decreasing rate was speedier for pristine CsPbBr_3_ NCs than CsPbBr_3_@Cu-BTC composite. The mechanism of PL intensity decline can be discussed for both samples. In pristine CsPbBr_3_ NCs, intensity decreases speedily due to the fast aggregation rate in toluene solution and decomposition of CsPbBr_3_ NCs to their precursors. Interestingly, the red shift in emission spectra of aging pristine CsPbBr_3_ NCs is directly related to the formation of trap states or defects, which can reduce the emission performance of NCs. Notably, in the case of protected CsPbBr_3_ NCs with the comparison of pristine CsPbBr_3_ NCs, very few emission intensity changes are observed. Cu-BTC inhibits the rate of aggregation and degradation of NCs, passivating trap states, or defects. These results indicate that the Cu-BTC MOF-treated CsPbBr_3_ NCs exhibit better long-term stability than pristine CsPbBr_3_ NCs.

Powder X-ray diffraction (PXRD) patterns of CsPbBr_3_ NCs, Cu-BTC, and CsPbBr_3_ NCs@Cu-BTC composite were recorded as shown in Fig. S2. For CsPbBr_3_ NCs, diffraction peaks were observed at 2θ = 15.27°, 21.49°, 30.68°, 34.38°, 37.79°, and 43.71° indexing to the (100), (110), (200), (210), (211), and (220) reflections, respectively. This XRD pattern suggests a cubic phase of as-synthesized CsPbBr_3_ NCs^34^. XRD pattern of Cu-BTC is similar to the previously reported in the literature^28,35^. The experimental PXRD pattern of Cu-BTC was readily indexed with cubic space group Fm-3 m and a = 26.434 Ǻ (CCDC Cif 7107285) as shown in Fig. S3^36^. The minor additional peaks were obtained which may arise due to CuO impurities as reported in previous research. The peak intensity of the diffraction pattern may be affected by the environmental moisture during analysis^37^. The presence of CsPbBr_3_ in the MOF can be ensured by observing the XRD pattern of the CsPbBr_3_ NCs@Cu-BTC composite. In this diffraction pattern, the peaks of both CsPbBr_3_ and Cu-BTC MOF are observed. Most of the peaks of CsPbBr_3_ are overlapped with Cu-BTC peaks, but the peaks at 2θ = 15.27° and 37.79° corresponding to the (100) and (211) planes of the cubic CsPbBr_3_ appeared. From these results, it is confirmed that CsPbBr_3_ NCs were incorporated and well dispersed in Cu-BTC MOF. The stability test of storage samples immersed in toluene was examined by XRD up to 60 days (Fig. S4). The XRD pattern of pristine CsPbBr_3_ NCs after aging shows a new peak at 12.81° with increasing intensity from 15 to 60 days, which can occur due to partial formation of Cs_4_PbBr_6_ as an impurity in solution which may affect the emission^38^.

The peak height of cubic CsPbBr_3_ gradually reduces up to 60 days, increasing sharpness. These changes in XRD spectra indicate the aggregation and degradation of CsPbBr_3_ NCs consistent with PL results^13^. After storage, the peak at 30.68° become split which indicates the transformation of cubic phase to orthorhombic phase lead to the phase distortion of CsPbBr_3_ NCs. This is also a reason for losing its optical performance after storage^39^.

The aggregation of CsPbBr_3_ NCs in toluene solution proved by PXRD analysis as well as TEM studies after 60 days as shown in Fig. S4. In PXRD results, the diffraction peaks become narrower after 60 days as compared to fresh sample diffraction pattern indicating the aggregation of CsPbBr_3_ NCs^40^. Moreover, the TEM image of CsPbBr_3_ NCs after 60 days in Fig. S4 is showing aggregation of NCs which consistent with PXRD and PL results of storage CsPbBr_3_ NCs. The storage CsPbBr_3_ NCs@Cu-BTC composite shows an almost similar XRD pattern after 60 days in Fig. S5, which suggests the superior stability of CsPbBr_3_ NCs with Cu-BTC MOF than pristine CsPbBr_3_NCs.

**Figure 4:**
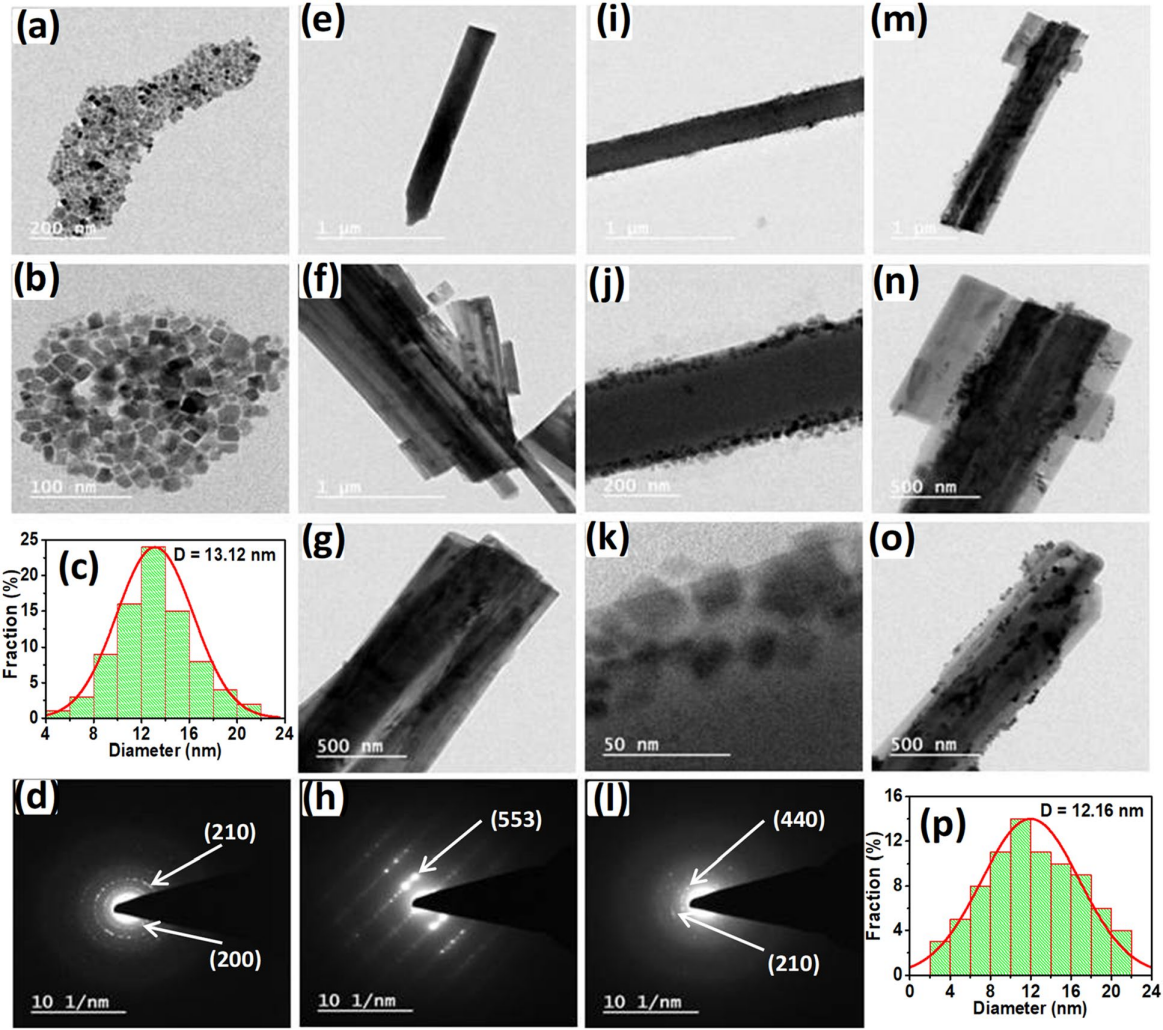
(**a**) TEM images of the as-prepared CsPbBr_3_ NCs, scale bar, 200 nm. (**b**) TEM images of CsPbBr_3_ NCs, scale bar, 100 nm. (**c**) Histogram of CsPbBr_3_ NCs particle size distribution. (**d**) SAED pattern of CsPbBr_3_ NCs. (**e**,**f**) TEM images of Cu-BTC MOF, scale bar, 1 µm. (**g**) TEM image of Cu-BTC MOF, scale bar, 500 nm. (**h**) SAED pattern of Cu-BTC MOF microrods. (**i**) TEM image of CsPbBr_3_@Cu-BTC composite, scale bar, 1 µm. (**j**) TEM image of CsPbBr_3_@Cu-BTC composite, scale bar, 200 nm. (**k**) TEM image of CsPbBr_3_@Cu-BTC composite, scale bar, 50 nm. (**l**) SAED pattern of CsPbBr_3_@Cu-BTC composite. (**m**) TEM image of CsPbBr_3_@Cu-BTC composite, scale bar, 1 µm. (**n**,**o**) TEM images of CsPbBr_3_@Cu-BTC composite, scale bar, 500 nm, with the bunch of microrods. (**p)** Histogram of CsPbBr_3_ NCs particle size distribution dispersed over Cu-BTC MOF microrods.

The morphology of the as-prepared CsPbBr_3_ NCs, Cu-BTC MOF, and engaged CsPbBr_3_ NCs with Cu-BTC MOF was investigated by transmission electron microscopy (TEM) analysis. As shown in Fig. 4a,b, the as-prepared CsPbBr_3_ shows nanoplate shape, with an average size of 13.12 nm (Fig. 4c), and the selective area electron diffraction (SAED) pattern of CsPbBr_3_ NCs in Fig. 4d represents (200) and (210) reflection planes of the cubic structure of CsPbBr_3_. Figure 4e–g exhibit microrods morphology of Cu-BTC MOF. Figure 4h shows a highly crystalline SAED pattern of MOF in which a crystal face (553) was observed that was consistent with its characteristic diffraction pattern. Figure 4i–k and m–o show TEM images of CsPbBr_3_@Cu-BTC after loading CsPbBr_3_ NCs solution into Cu-BTC MOF powder. No bare CsPbBr_3_ particles appeared in all the TEM images of CsPbBr_3_@Cu-BTC, which unfolded the distribution of CsPbBr_3_NCs with the surface of Cu-BTC MOF microrods. Figure 4k clearly shows that the CsPbBr_3_ NCs are strewn over the Cu-BTC microrods. Moreover, the SAED pattern for CsPbBr_3_@Cu-BTC exhibits (210) and (440) crystal facets, in which (210) represents the cubic CsPbBr_3_ and (440) is characteristic for Cu-BTC, as shown in Fig. 4l. The average particle size of CsPbBr_3_ is 12.16 nm in the CsPbBr_3_@Cu-BTC composite (Fig. 4p). Meanwhile, the size of CsPbBr_3_ decreased slightly in composite, which may be due to the surface passivation of NCs by BTC.

**Figure 5:**
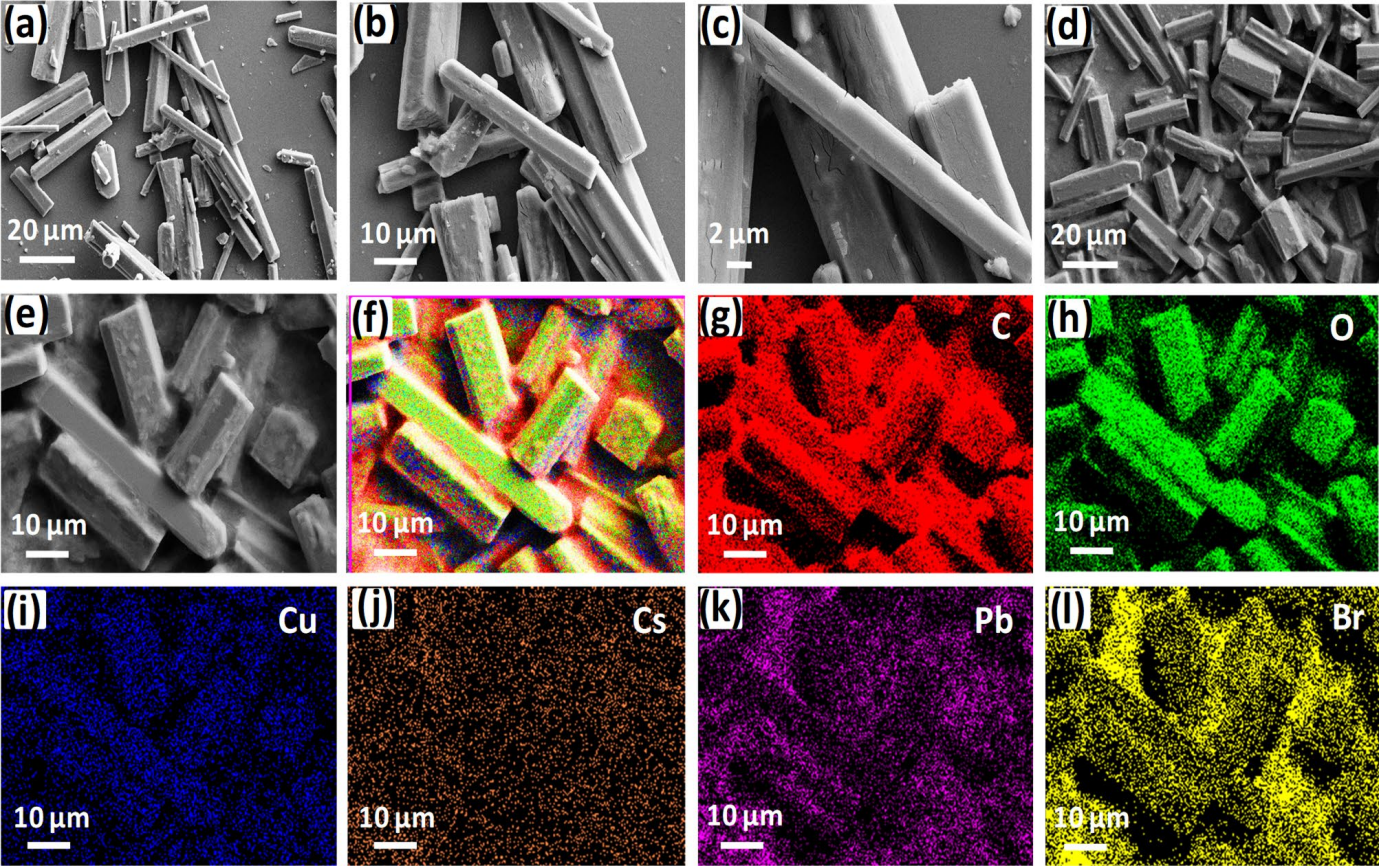
(**a–c**) Typical SEM images of Cu-BTC MOF at different magnifications. (**d**,**e**) SEM images of CsPbBr_3_@Cu-BTC nanocomposites at different magnifications. (**f**) A combined elemental mapping image of all constituent elements in CsPbBr_3_@Cu-BTC nanocomposites, including (**g**) C, (**h**) O, (**i**) Cu, (**j**) Cs, (**k**) Pb, and (**l**) Br, individual elemental analysis.

Further, the morphology of Cu-BTC MOF and CsPbBr_3_@Cu-BTC composite was observed by scanning electron microscopy (SEM), as  shown in Fig. 5. The SEM images in Fig. 5a–c display microrods shape of Cu-BTC at different magnifications. When CsPbBr_3_ NCs are loaded into Cu-BTC MOF, the SEM images for CsPbBr_3_@Cu-BTC composite, as shown in Fig. 5d,e, represent a similar morphological pattern as Cu-BTC MOF. Figure 5f–l show the elemental mapping of synthesized CsPbBr_3_@Cu-BTC composite. In these results, the existence of Cs, Pb, and Br elements with C, O, and Cu indicates the uniform distribution of CsPbBr_3_ NCs in Cu-BTC MOF.

The composition of elements was identified by energy-dispersive x-ray (EDX) analysis for Cu-BTC and CsPbBr_3_ in Cu-BTC MOF. The EDX spectra (Fig. S6) at an arbitrary point of microrods show C, O, and Cu signals, indicating the distribution of these elements in Cu-BTC MOF construction. The CsPbBr_3_@Cu-BTC composites at a random point of microrods, the additional Cs, Pb, and Br signals are also obtained with the C, O, and Cu in the atom ratio of 1:1:3, which show the presence of CsPbBr_3_ NCs (Fig. S7). These results illustrate the distribution of CsPbBr_3_ NCs throughout the Cu-BTC microrods.

The porous behaviour of samples was investigated by Brunauer Emmett-Teller (BET) gas-sorption measurements. The N_2_ adsorption–desorption isotherms of Cu-BTC and CsPbBr_3_@Cu-BTC composite as shown in Fig. S8 are revealing micropore nature. The pore size distribution was calculated by using the Barrett–Joyner–Halenda (BJH) method. The curves of pore size distribution as shown in Fig. S8b and d exhibit microporous range. The surface area and pore volume for Cu-BTC were calculated 821.30 m^2^/g and 0.461 cm^3^/g and for CsPbBr_3_@Cu-BTC composite 565.73 m^2^/g and 0.316 cm^3^/g, respectively. The decreasing surface area and pore volume in CsPbBr_3_@Cu-BTC composite clearly indicate the incorporation of CsPbBr_3_ NCs with Cu-BTC^41^. In this report, the obtained pore volume and surface area of Cu-BTC are almost similar to Ahmed et al.^42^ and Peedikakkal et al.^43^ reports.

FTIR studies were carried out as shown in Fig. S9. The surface binding ligands (oleic acid and oleylamine) in CsPbBr_3_ NCs were disclosed by their characteristic vibrational signals. The vibrational peak at 2922 cm^−1^ reveals the stretching vibration of the C–H bond in –CH_3_ and the peak of 2856 cm^−1^ is due to the C–H bond in –CH_2_ of the aliphatic hydrocarbon chain. The peak of 1629 cm^−1^ is due to the N–H bending vibration for the NH_2_ group of oleylamine. Moreover, the peak at 1563 cm^-1^ is due to the stretching vibration in –COO of oleic acid. In this contrast, for Cu-BTC, the peak at 741 cm^-1^ attributes to the Cu–O bond, which confirms the metal–ligand coordination. A band at around 1619 cm^-1^ indicates the symmetric stretching of C=O groups in BTC, and the peak at 1380 cm^-1^ represents C=C of benzene. These all characteristic vibrational signals confirm the formation of Cu-BTC^44^. The vibrational spectra for CsPbBr_3_ NCs@Cu-BTC composite, the surface ligand peaks of CsPbBr_3_ NCs cannot be differentiated due to the strong overlapping of Cu-BTC peaks.

XPS analytical technique was performed to identify the chemical compositional elements of CsPbBr_3_@Cu-BTC compared with Cu-BTC MOF and CsPbBr_3_ as shown in Fig. S10. For Cu-BTC, the spectrum only shows the binding energy peaks of C1s, O1s, and Cu2p, which are characteristic peaks for Cu-BTC MOF. The XPS spectrum of CsPbBr3@Cu-BTC exhibits additional peaks of Cs3d, Pb4f., and Br3d with C1s, O1s, and Cu2p binding energy peaks, which suggest the formation of CsPbBr_3_@Cu-BTC composite. It is worth noting that the negative binding energy shift of Pb4f. was observed for CsPbBr_3_@Cu-BTC compared to pristine CsPbBr_3_ (Fig. S11), which may be due to the strong interaction of Pb^2+^ with BTC ligand^45^.

The optical performance of CsPbBr_3_ NCs has been improved by preventing its degradation and aggregation using a variety of surface ligands^16,46^. In the CsPbBr_3_@Cu-BTC composite, the oxygen atoms of the BTC linker present in the MOF may bind the CsPbBr_3_ NCs, which results from the emergence of highly stable CsPbBr_3_ NCs with low surface defects. The strong interaction of CsPbBr_3_ NCs with Cu-BTC MOF is supported by the TEM images. However, the UV light field microscopic images show the high fluorescent surface decorated CsPbBr_3_@Cu-BTC composite.

## Conclusion

In summary, we showed the experimental realization of as-synthesized bright green luminescent CsPbBr_3_@Cu-BTC composite at ambient conditions. Cu-BTC MOF was used as a host to protect the degradation of CsPbBr_3_ NCs in the solution system. Moreover, the obtained CsPbBr_3_@Cu-BTC exhibited PLQY of 96.51% with excellent luminescent property relative to that of 51.66% of pristine CsPbBr_3_ NCs. Therefore, our approach significantly improves the long-term stability and optical properties of perovskite NCs by forming composite materials.

## Experimental details

### Chemicals

Cesium bromide, lead (II) bromide,copper (II) nitrate trihydrate, oleic acid, and oleylamine were purchased from Sigma Aldrich. Toluene, ethanol, and N,N-dimethylformamide (DMF) were obtained from Thomas Baker. 1,3,5-Benzene tricarboxylic acid (BTC) was purchased from TCI. All the chemicals were used as received without further purification.

### Synthesis of CsPbBr_3_ NCs

In a typical preparation, 0.1 mmol of CsBr and 0.1 mmol of PbBr_2_ were dissolved in 2 mL DMF then 80 µL oleylamine and 250 µL oleic acid were added with vigorous stirring. After that, a 200µL of precursor solution was dispersed into a 3 mL toluene solvent. Finally, we observed green luminescence of CsPbBr_3_ NCs under UV light (365 nm).

### Synthesis of Cu-BTC MOF

4.1 mmol (1 g) of Cu(NO_3_)_2_.3H_2_O was dissolved into 30 mL of water, and 2.5 mmol (0.53 g) of 1,3,5-Benzene tricarboxylic acid was dissolved in 30 mL ethanol. After that, the copper nitrate solution was added dropwise into the BTC solution under vigorous stirring for 15 min at room temperature. The sky blue-colored precipitate was found. Then, the precipitate was filtered and washed with ethanol and water  for 3 times. The powder form was obtained after drying at 80 °C under vacuum overnight.

### Synthesis of CsPbBr_3_@Cu-BTC nanocomposite

For the typical synthesis of perovskite NCs@MOFs nanocomposites, 40 mg of Cu-BTC powder was added into 3 mL CsPbBr_3_ NCs containing toluene solution and sonicated for 5 min. A green-colored solution was obtained. We observed bright green luminescence under UV light (365 nm).

### Instrumentation

Shimadzu UV–Vis 2450 Spectrophotometer was used for recording the UV–Vis absorption spectra in the range of 460–600 nm. Photoluminescence spectra were performed by using Horiba Scientific Fluoromax-4C Spectrophotometer. A quartz cuvette of 10 mm path length and volume of 3 ml was used forcollecting the spectra.Quantum yield as an absolute quantum yield was measured directly by using Edinburgh instruments FLS 980. Powder XRD was carried out in a Bruker-D8 using Cu-Ka radiation with an accelerating voltage of 40 kV from 5° to 50° with a rate of 1^°^/min. The thin film samples were prepared on silica glass.Transmission electron microscopy (TEM) studies were carried out using TECHNAI G2 20 S-TWIN. These were performed by taking a drop of highly diluted sample on a carbon-coated copper grid and drying it at 80 °C under vacuum overnight before analysis. Field emission scanning electron microscopy (FESEM) images were recorded for all the samples by Carl Zeiss Ultra Plus. The thin film samples were prepared on silica glass and drying it at 80 °C under vacuum overnight. N_2_ adsorption–desorption isotherms were measured using a Micromeritics ASAP 2020 adsorption analyzer at 77 K. Fourier transform infrared spectroscopy (FTIR)spectra of all the samples were recorded by using Thermo Scientific Nicolet 6700. X-ray photoelectron spectroscopy (XPS) analysis of the thin film of samples prepared on silica glass was studied using PHI 5000 Versa Probe III.

## Supplementary Information

Supplementary Figures.
