# Supplementary material for: Significant boost of the stability and PLQYof CsPbBr3 NCs by Cu-BTC MOF
Source: Sci Rep. 2022 May 12;12:7848. doi: 10.1038/s41598-022-11874-6 (PMC9098410; doi:10.1038/s41598-022-11874-6)
Supplement: Supplementary file 1 — Supplementary Figures. [file 41598_2022_11874_MOESM1_ESM.docx]

**Supporting Information**

**Significant Boost of the Stability and PLQYof CsPbBr_3_ NCs by Cu-BTC MOF**

Hari Shankar^a^, William W. Yu^b^,Youngjong Kang^c^and Prasenjit Kar^a^*

^a^ Department of Chemistry, Indian Institute of Technology Roorkee, Uttarakhand- 247667, India

^b^Department of Chemistry and Physics, Louisiana State University, Shreveport, Louisiana 71115, United States

^c^Department of Chemistry, College of Natural Sciences, Hanyang University, 222 Wangsimni-ro, Seongdong-gu, Seoul, 04763, Korea

Email:kar.prasen@gmail.com, prasenjit.kar@cy.iitr.ac.in

**Figure S1** Optical microscopic images of CsPbBr_3_@Cu-BTC composite under a) bright field and b) UV- light field.

**Figure S2** XRD patterns of the as-prepared CsPbBr_3_ NCs, Cu-BTC MOF, and CsPbBr_3_@Cu-BTC composite.

**Figure S3** XRD pattern of synthesized Cu-BTC with simulated XRD pattern of Cu-BTC

**Figure S4** a) XRD patterns of the as-prepared CsPbBr_3_ NCs up to 60 days. b) TEM image of CsPbBr_3_ NCs after 60 days.

**Figure S5** XRD patterns of the as-prepared CsPbBr_3_ NCs, Cu-BTC MOF, and CsPbBr_3_@Cu-BTC composite after 60 days.


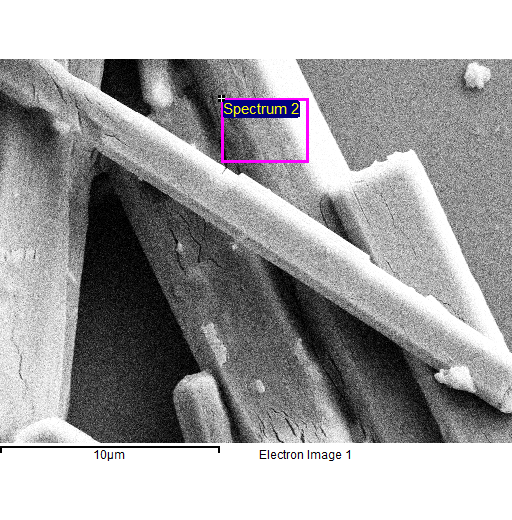


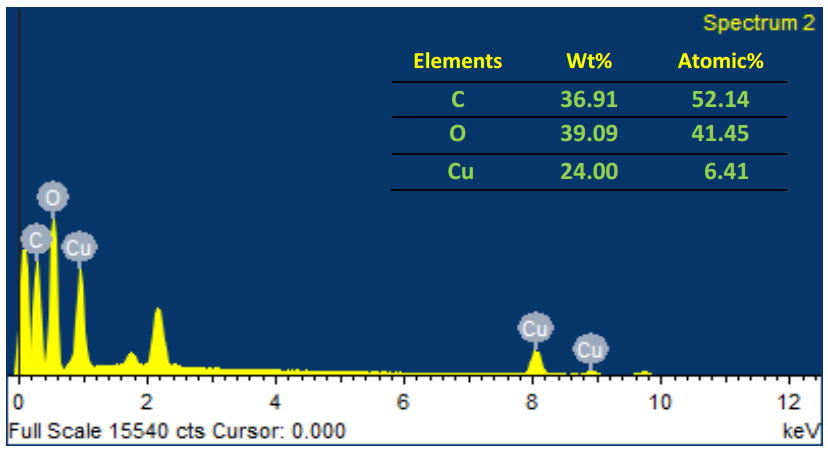


**Figure S6** SEM-EDX analysis of Cu-BTC MOF, showing the elemental composition of C, O, and Cu.


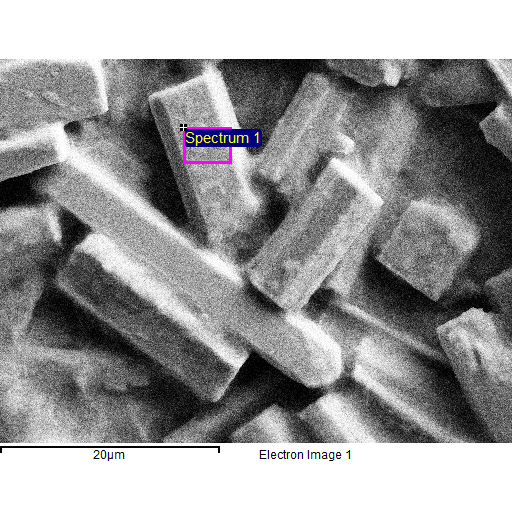


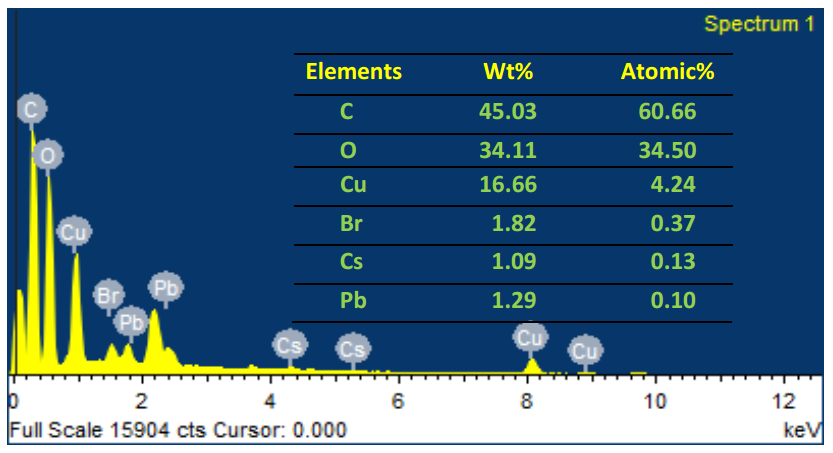


**Figure S7** SEM-EDX analysis of CsPbBr_3_@Cu-BTC composite, showing the elemental composition of C, O, and Cu for Cu-BTC with the composition of Cs, Pb, and Br for CsPbBr_3_ NCs.

**Figure S8** a) N_2_ Adsorption-desorption isotherm of Cu-BTC. b) Pore size distribution curve of Cu-BTC. c) N_2_ Adsorption-desorption isotherm of CsPbBr_3_@Cu-BTC composite. d) Pore size distribution curve of CsPbBr_3_@Cu-BTC.

**Figure S9** FTIR spectra of the as-prepared CsPbBr_3_ NCs, Cu-BTC MOF, and CsPbBr_3_@Cu-BTC composite.

**Figure S10** XPS spectra of the as-prepared CsPbBr_3_ NCs, Cu-BTC MOF, and CsPbBr_3_@Cu-BTC composite.

**Figure S11** XPS spectra of Pb of the as-prepared CsPbBr_3_ NCs and CsPbBr_3_@Cu-BTC composite.
